# 

**Published:** 1873-04-01

**Authors:** 


American Medical Association.—The next annual meeting of this national organization will be held in St. Louis, commencing on Tuesday morning, May 6th, 1873. All permanently organized medical societies are entitled to one delegate for every ten of their resident members. We hope the profession, especially in the West and Northwest, will be fully represented. Will the several societies in this city and State see that their delegates are appointed and duly accredited in good time?

Association of American Medical Editors.—In accordance with a resolution adopted at the last meeting, this Association will hold its next annual meeting in St. Louis, Mo., on the evening of Monday, May 5th, commencing promptly at 8 o'clock. Notice of the particular place of meeting will be given by the Secretary in due time.

